# Editorial: Open Abdominal Treatment: How Much Evidence Do We Have?

**DOI:** 10.3389/fsurg.2021.696296

**Published:** 2021-06-21

**Authors:** Arnulf Gregor Willms, Rene' H. Fortelny, Frederik Berrevoet, Ulrich A. Dietz, Ulf Petersson, Robert Schwab

**Affiliations:** ^1^Department of General-, Visceral- and Thoracic Surgery, German Armed Forces Central Hospital Koblenz, Koblenz, Germany; ^2^Department of General, Viszeral and Oncologic Surgery, Wilhelminenspital, Vienna, Austria; ^3^Department for General and HPB Surgery and Liver Transplantation, Ghent University Hospital, Ghent, Belgium; ^4^Department of Visceral, Vascular and Thoracic Surgery, Cantonal Hospital of Olten, Olten, Switzerland; ^5^Department of Surgery, Skane University Hospital, Malmö, Sweden; ^6^Faculty of Medicine, Lund University, Lund, Sweden

**Keywords:** open abdomen, peritonitis, abdominal trauma, abdominal compartment syndrome, vacuum therapy

Open abdominal treatment (OAT) is a surgical therapy strategy for critically ill patients with serious intra-abdominal pathologies. Since its introduction about 30 years ago, it has had a permanent place in trauma and damage control surgery, but also in treatment of visceral surgical emergencies. Applied to the right patient, the OAT strategy has been shown to reduce morbidity and mortality in patients whose systemic compensation mechanisms and physiological reserves (circulation, blood coagulation, etc.) are almost exhausted due to the serious intra-abdominal pathology.

This procedure and core topic of this issue is the result of a paradigm shift in emergency abdominal surgery. The aim of the OAT is to minimize the initial, surgical-related secondary damage through a shortened initial operation and a temporary abdominal wall closure. However, these patients then necessarily need one or more further surgical interventions. Thus, the initiation of the OAT represents a decisive course and the subsequent management an enormous logistical and medical challenge: Patients with OAT need a structured therapy concept with close coordination of surgery and intensive care medicine in order to benefit from the advantages of the procedure. Advantages include the shortened operating time for index surgery, the easy re-evaluation (second look), the possibility of repeated decontamination (lavage), a better ventilation situation, as well as an improvement in renal and intestinal perfusion.

Nevertheless, the OAT course is associated with serious potential disadvantages. The most elementary possible negative consequences are: the formation of entero-atmospheric fistulas, the lateral retraction of the abdominal wall fascia, which makes a later fascia closure much more difficult, and fluid losses through exposure of the viscera.

The present edition spans a wide range and reflects not only current knowledge but also new strategies and approaches. The attempt is made to map all important therapy goals, such as fascia traction and fistula prevention as well as hernia prophylaxis. This issue includes reports of many years of experience of a center with vacuum therapy as one of the most important and established therapy elements with a large patient population as well as new technologies for prophylactic mesh implantation with the aim of hernia prophylaxis on only a few patients. There are excellent descriptions of patient subgroups such as pancreatitis or experiences with abdominal compartment syndrome in ECMO patients. There are very interesting experimental works on the suction effect as well as review articles on important topics such as fistula prevention and dynamic fascia traction and, last but not least, on the important parameter, the outcome after treatment.

We wish you a lot of fun and knowledge while reading and would like to thank all authors for the successful cooperation.

## Author Contributions

All authors listed have made a substantial, direct and intellectual contribution to the work, and approved it for publication.

## Conflict of Interest

The authors declare that the research was conducted in the absence of any commercial or financial relationships that could be construed as a potential conflict of interest.

